# Microbial transformation from normal oral microbiota to acute endodontic infections

**DOI:** 10.1186/1471-2164-13-345

**Published:** 2012-07-28

**Authors:** William WL Hsiao, Kevin L Li, Zhenqiu Liu, Cheron Jones, Claire M Fraser-Liggett, Ashraf F Fouad

**Affiliations:** 1 University of Maryland School of Medicine, Institute for Genome Sciences, Baltimore, MD, 20201, USA; 2 University of Maryland School of Dentistry, Department of Endodontics, Prosthodontics and Operative Dentistry, Baltimore, MD, 20201, USA; 3 University of Maryland School of Medicine, Department of Epidemiology and Preventive Medicine, Baltimore, MD, 20201, USA

**Keywords:** Endodontic infection, Endodontic microbiome, Periapical abscess, Oral microbiota, Next generation sequencing, 16S rRNA gene, Bacterial diversity

## Abstract

**Background:**

Endodontic infections are a leading cause of oro-facial pain and tooth loss in western countries, and may lead to severe life-threatening infections. These infections are polymicrobial with high bacterial diversity. Understanding the spatial transition of microbiota from normal oral cavities through the infected root canal to the acute periapical abscess can improve our knowledge of the pathogenesis of endodontic infections and lead to more effective treatment. We obtained samples from the oral cavity, infected root canal and periapical abscess of 8 patients (5 with localized and 3 with systemic infections). Microbial populations in these samples were analyzed using next-generation sequencing of 16S rRNA amplicons. Bioinformatics tools and statistical tests with rigorous criteria were used to elucidate the spatial transition of the microbiota from normal to diseased sites.

**Results:**

On average, 10,000 partial 16S rRNA gene sequences were obtained from each sample. All sequences fell into 11 different bacterial phyla. The microbial diversity in root canal and abscess samples was significantly lower than in the oral samples. *Streptococcus* was the most abundant genus in oral cavities while *Prevotella* and *Fusobacterium* were most abundant in diseased samples. The microbiota community structures of root canal and abscess samples were, however, more similar to each other than to the oral cavity microbiota. Using rigorous criteria and novel bioinformatics tools, we found that *Granulicatella adiacens, Eubacterium yurii, Prevotella melaninogenica, Prevotella salivae, Streptococcus mitis,* and *Atopobium rimae* were over-represented in diseased samples.

**Conclusions:**

We used a novel approach and high-throughput methodologies to characterize the microbiota associated normal and diseased oral sites in the same individuals.

## Background

Endodontic infections are a leading cause of oro-facial pain, localized and spreading dental infections and loss of teeth. It is known that endodontic infections are the leading cause of tooth loss in western countries [1]. The most recent figures available indicate that about 15 million primary endodontic treatment procedures were performed in the U.S. in 2005-2006 [2]. Approximately 34% of all presenting endodontic cases have an infection with periapical lesions [3]. Extrapolating these data to the national figure, about 5.1 million primary endodontic infections are treated in the U.S. every year.

The etiology of endodontic infections is heterogeneous and is likely to be polymicrobial[4]. The endodontic pathogens are most likely introduced from deep caries, and/or sulcular and periodontal bacteria. These infections are unique in that a subgroup of oral microorganisms populates a previously sterile space, are fairly protected from outside influences and cause a disease process that ranges from mild discomfort to severe, life-threatening infections. The success of endodontic treatment and the survival of the tooth are significantly reduced in cases with infection compared with treating the inflamed vital pulp [3,4]. There are profound patient and societal impact that arises from the morbidity involved and the cost of the management of non-healed cases. About 56% of all non-traumatic dental emergencies are in the form of a periapical abscesses and toothaches [5]. Furthermore, microbial invasion of the periapical lesion and systemic circulation during pathogenesis of the disease, during treatment or during the healing phase may cause or contribute to significant systemic diseases. The infection can spread to cause mediastinitis [6,7], fatal necrotizing fasciitis [8] or brain abscess [9]. In 2007, 7,886 hospitalizations were attributed to periapical abscesses [10].

Due to the polymicrobial nature of the disease, a survey of the oral microbial community (or microbiota) is necessary to further our understanding of the infection. Several studies to date have characterized the human oral microbiome (genes from the microbiota) under healthy conditions to establish a baseline (or core) oral community [11-13]. Several observations have emerged from these analyses. It is clear that while only a small number of bacterial phyla (approximately 9-13 out of the 39 phyla in RDP Database -http://rdp.cme.msu.edu/) have been observed in human oral samples, diversities at the species and strain level account for the majority of the individual variations [12]. In other words, at a broad categorization, there are a limited number of bacterial taxa which inhabit the human oral cavity, but at a finer scale, the oral cavity appears to be colonized by a diverse and variable set of bacterial strains. This observation suggests that each individual is his or her own most suitable control for establishing his or her baseline microbiome. It has also been observed that certain taxa are prevalent in healthy human oral cavities. These include *Streptococcus, Veillonella, Granulicatella, Neisseria, Haemophilus, Corynebacterium, Rothia, Actinomyces, Prevotella, Capnocytophaga, Porphyromonas,* and *Fusobacterium*[11,13]. However, it can not be ruled out that some of these genera contain opportunistic pathogens that can cause diseases in previously sterile oral sites, such as the root canal system. Moreover, while some species are present in multiple oral sites, each of the sampled sites has characteristic microbiome profiles and harbors subsets of the overall oral microbiome, probably reflecting the micro-environment of each site [11,13]. Another observation is that by using high-throughput next-generation sequencing technology, the species richness of oral microbiota is significantly greater than previous estimations using low coverage sampling methods [13-15]. With deeper sequence coverage, many novel species have been uncovered. Sampling depth is therefore critical for gaining a more complete picture of the oral microbial community. Besides the healthy oral microbiota, several studies have looked at the microbiomes of specific oral diseases. These studies demonstrated that diseased sites harbor different bacteria compared to the healthy microbiome. Moreover, micro-environments can exist in diseased sites which can affect treatment efficacy [16]. Opportunistic pathogenic genera such as *FusobacteriumTreponemaTannerella Porphyromonas *and *Prevotella *are more prevalent in diseased samples [17-19]. However, no single organism has been reported to consistently associate with specific diseases. Conversely, bacteria that have been associated with oral diseases have also been observed in healthy subjects [20,21].

Endodontic infections take place in a closed, stable, relatively controlled environment that lends itself well to sampling and analysis. However, previous research that sought to analyze microbial pathogens in endodontic infections has yielded incomplete and non-conclusive data on the associations of these infections with peri-operative pain and flare-ups, swelling and systemic invasion, and persistence of disease after treatment. The most likely reasons for this lack of adequate information on microbial associations in aggressive endodontic infections are: lack of establishment of a base-line oral microbiome for the patients sampled, examination of only few selected bacteria as sole organisms in these infections, use of techniques that do not provide sufficient depth of coverage of microbial diversity present, and the examination of specific microbial taxa without consideration of the virulence genes and proteins that may be expressed.

It is believed that in patients who have primary endodontic pathosis without other oral disease, bacteria migrate from normal oral tissues to the necrotic pulp, may undergo compositional and numerical shifts and initiate periapical inflammation which is frequently associated with moderate to severe symptoms. Therefore, in the same individual who does not have evidence of periodontal or other oral disease, but has evidence of primary endodontic pathosis, the changes in microbial ecology from health to disease states can be simultaneously studied. To address the shortcomings of previous studies, our study combined sampling of both diseased and non-diseased sites within the same individuals and the application of next generation high throughput sequencing to generate deep sequencing coverage. The aim of this study was to elucidate the spatial transition of microbial populations from the normal oral cavity through the infected root canal to the acute periapical abscess.

## Results and discussion

In this study, sampling of microbiota from healthy oral sites, as well as two endodontically diseased sites in each patient was performed, in order to study the transformation of the microbiome from health to disease. It is recognized that the oral sites may have undergone significant changes from the time of initial endodontic infection until the time of sampling, and therefore, these samples could be considered cross-sectional. However, in the absence of the ability to sample this disease at different times without treatment in patients, and inability to know with precision when infection actually begins in these cases, we reasoned that this approach would be a reasonable alternative for longitudinal or temporal sampling of endodontic infections.

### DNA extraction and sequencing

The measured DNA concentrations from samples variedfrom 15.8ng/ul to 394.7ng/ul for oral samples (OS), from 6.5ng/ul to 734.1ng/ul for root canal samples (RC), and 13.6ng/ul to 4503.20ng/ul for abscess samples (AS) (see Table 1 for sample summary). The large variation likely reflects the amount of primary samples collected and host DNA contamination. When the level of contaminant was too high, PCR amplification with broad-range 16S primers would fail and the amount of template DNA has to be lowered resulting in lower amount of final products. When possible, equal molar amount of barcoded PCR products (100ng) from each sample was pooled for sequencing. In 4 cases (E007AS, E007RC, E010AS, and E016AS), lower amounts (ranges from 61ng to 93ng) were pooled due to insufficient PCR amplification. In one case (E009AS), no PCR products could be obtained from the primary sample. As a result, 23 samples were used for sequencing. In total, 231,519 sequences, which passed the quality filters and were successfully assigned to a sample based on unique barcodes, were generated from these samples. Because there were other samples sequenced in the same 454 sequencing run, we could not report the number of sequences belonging to this study prior to binning. However, the overall quality of the sequencing run was good with over 505,000 high quality reads generated (50.02% of the reads pass 454’s quality filter; average quality: 37.99; quality standard deviation: 4.94) and a median length of 362 bases (range: 200nt to 1166nt; standard deviation 29.72) from half of a plate. The breakdown of the number of sequences in each sample is shown in Table 1 and in Additional file 1. There was an average of 10,516 reads from each of the OS samples, 10,481 reads from each of the RC samples, and 9,078 reads from each of the AS samples. While there were some coverage variations among samples, the average numbers of reads from each of the oral sites were roughly comparable. The number of reads from each sample roughly corresponds to the amount of calculated input PCR products. The abscess samples with low PCR yields also resulted in fewer reads.

**Table 1:** DNA extraction, PCR amplification and sequencing results

**Sample**	**Systemic or Local**	**DNA Concentration (ng/ul)**	**Total Volume (ul)**	**Total DNA (ug)**	**PCR Amplification**	**Number of Sequencing Reads**
E007 AS	Local	192.40	42.00	8080.80	successful	7581
E007 OS	Local	20.50	33.00	676.50	successful	10927
E007 RC	Local	9.20	12.00	110.40	low PCR yield (84.21ng)	8791
E009 AS	Systemic	13.60	25.00	340.00	no amplification	n/a
E009 OS	Systemic	33.60	32.00	1075.20	successful	11876
E009 RC	Systemic	46.40	37.00	1716.80	successful	11319
E010 AS	Systemic	1561.80	36.00	56224.80	low PCR yield (61.08ng)	4277
E010 OS	Systemic	126.60	34.00	4304.40	successful	9435
E010 RC	Systemic	21.00	35.00	735.00	successful	11504
E012 AS	Local	4503.20	42.00	189134.40	low PCR yield (93.39ng)	5800
E012 OS	Local	394.70	35.00	13814.50	successful	11211
E012 RC	Local	67.30	40.00	2692.00	successful	11835
E016 AS	Local	15.50	27.00	418.50	low PCR yield (80.46ng)	6774
E016 OS	Local	19.60	27.00	529.20	successful	12651
E016 RC	Local	6.50	5.00	32.50	successful	11809
E017 AS	Local	199.90	40.00	7996.00	successful	10735
E017 OS	Local	22.00	35.00	770.00	successful	11305
E017 RC	Local	128.20	40.00	5128.00	successful	10365
E018 AS	Systemic	727.20	40.00	29088.00	successful	11439
E018 OS	Systemic	15.80	30.00	474.00	successful	10148
E018 RC	Systemic	23.50	31.00	728.50	successful	8601
E019 AS	Local	18.30	30.00	549.00	successful	16937
E019 OS	Local	16.50	27.00	445.50	successful	6575
E019 RC	Local	734.10	35.00	25693.50	successful	9624

### An overview of the bacterial taxa in the samples

**Figure 1:**
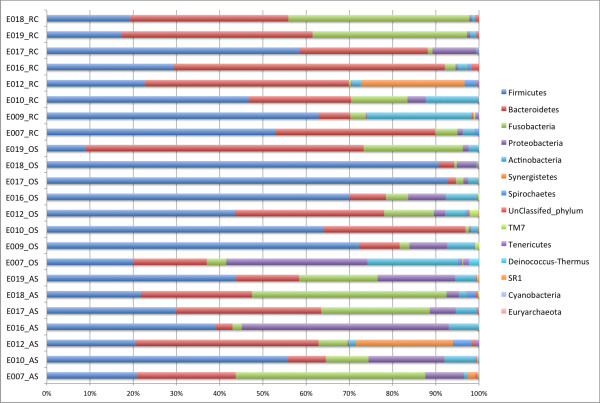
Distributions (relative abundances) of phyla in samples arranged by sample type (RC: root canal; OS: oral sample; and AS: abscess sample).

We first assign the reads to known taxa using RDP Classifier, a naïve Bayesian classifier based on characteristic oligonucleotide frequencies associated with each taxon, to get an overview of the taxonomic distribution of our samples [22]. About 0.3% of the sequences (696/231519)could not be classified at the phylum level with a 0.5 confidence threshold. BLAST searches of these sequences against HOMD database suggested that these were valid sequences that RDP Classifier failed to classify [23]. Eleven phyla were found in at least two of our samples. These were: Firmicutes, Bacteroidetes, Fusobacteria, Proteobacteria, Actinobacteria, Synergistetes, Spirochaetes, TM7, Tenericutes, Deinococcus-Thermus, and SR1. Organisms from all these phyla have been previously found in oral samples [24]. Two phyla, Euryarchaeota and Cyanobacteria were found in only one sample each. One and two sequences were assigned to phyla, Euryarchaeota and Cyanobacteria, respectively. These phyla were previously not found in oral samples. BLAST searches confirmed that these were mis-assignments by RDP Classifier. These 3 sequences were dropped from further statistical analyses. Figures 1 summarizes the distribution of the phyla in each sample. For breakdown of the different taxonomic ranks (phylum, class, order, family, and genus) by sample sites and by subjects, see Additional file 2 (Excel file). In all 3 sites, Firmicutes had the highest relative abundances (57.7% for oral samples, 38.7% for root canal samples, and 33.1% for abscess samples). The next most abundant phylum was Bacteroidetes (21.5% in oral samples, 36% in root canal, and 21.7% in abscess). In abscess samples, Fusobacteria were also highly abundant at 21.6%.

**Figure 2:**
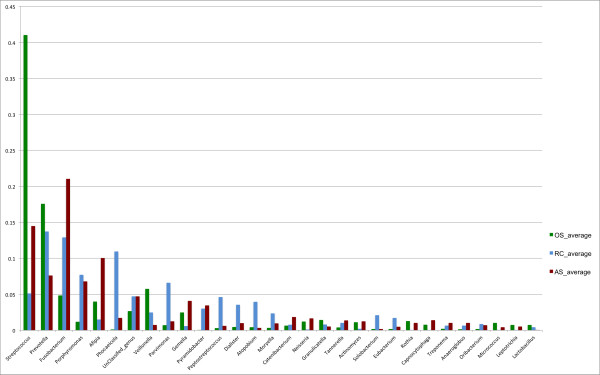
Relative abundance of phyla by sample sites (RC: root canal; OS: oral sample; and AS: abscess sample).

At the genus level, approximately 4% (9492/231519) of the sequences could not be classified at the 0.5 confidence threshold. The relative abundances of different genera changed from oral samples to root canal samples to abscess samples (Figures 2). Because the number of reads per sample was different, we first derived the relative abundances of each genus within each sample before averaging the results by site to obtain the average relative abundance of the genera by site. The most abundant genus in oral samples was *Streptococcus*. The most abundant genera in root canals were *Prevotella* and *Fusobacterium*. The most abundant genus in abscess was *Fusobacterium*. These observations match previously reported results in acute endodontic infections [19,25], but this is the first report to clearly demonstrate the shift in microbiota in the three sites (one normal and two diseased) in the same individuals at a single time point.

Each individual sample, on average, contained 68.9 known genera (range: 26 -132). The oral samples had on average 78.5 genera (range: 40-132). The root canal samples had on average 52.5 genera (range: 26-81). The abscess samples had on average 76.8 genera (range: 55-101). Nine genera commonly detected in oral specimens (*StreptococcusPrevotellaFusobacteriumPorphyromonasAfipiaParvimonasDialisterTannerella*, and *Eubacterium*) were identified in all of our samples. Another common oral microorganism *Treponema* was identified in 19 of 23 samples. Because the sampling depth of each sample varies and because the variances of the abundance species and rare species are different, we calculated the relative abundance and applied arcsine transformation to the proportions in order to stabilize the variance and to allow us to use standard statistical tests on the samples [26]. The relative abundances and the arcsine transformed values of all of the genera for each site are listed in Additional file 3. Using ANOVA and paired-t-tests, we compared taxa from one site to others to identify genera that were differentially distributed in one site with respect to another. By using paired-t tests and sampling multiple sites within the same individuals, we could establish the baseline normal microbiota for each individual and use that as a reference to analyze the microbiota of diseased sites. Comparing RC samples to OS samples, the relative abundance of genera *AbiotrophiaRothiaStreptococcusCapnocytophaga* and *Leptotrichia*were significantly lower in RC samples at p-value cutoff of 0.01. Comparing AS samples to OS samples, *Diaphorobacter* and *Collinsella*were significantly over-represented in AS samples. No genus was detected to be significantly different at p-value cutoff of 0.01 when comparing RC and AS. For completeness, Additional file 4 lists all differentially distributed genera with p-values < 0.05. It is important to note that genus level comparison may be too coarse to identify taxa that are associated with diseases since many of the above genera consist of pathogens and non-pathogens (e.g. *Prevotella *and *Streptococcus* have been found on both healthy and diseased oral tissues). Moreover, the differentially distributed genera detected with this approach tend to be low abundance organisms. Therefore, in the section below we also analyzed the differential distribution of Operational Taxonomic Units (OTUs) using 99% sequence identity as cutoff.

Since RDP Classifier could only classify a sequence down to the genus level, we used SpeciateIT to further classify the genera of interest to the species level [27]. SpeciateIT clusters sequences within a genus into Operational Taxonomic Units (OTUs) and compares them to reference sequences from type strains. OTUs that were closely related to an annotated species but distant from all other clusters were assigned to that species whereas other OTUs remain unnamed. Given that the 454 pyrosequences are shorter than full-length 16S genes, care is needed to assign a sequence to a species. Unlike commonly used “top BLAST hits” approach where a sequence is often “forced” to have an assignment, this approach allowed us to classify sequences down to the species level without over assigning more distantly related sequences to known species. First, we looked at the genera that were significantly differentially distributed in one site compared to another. The most abundant *Streptococcus* species found in all 3 sites was *S. mitis*. Other common oral species such as *S. alactolyticusS. anginosusS. cristatusS. gordoniiS. constellatusS. mutansS. oralisS. parasanguinisS. pneumoniaeS. salivarius*, and *S. sanguinis*, were also found in our OS samples. It is worth noting that species identification of the *Streptococcus* genus is difficult using 16S rRNA gene fragment sequences and several species such as *S. mitisS. pseudopneumoniae* and *S. pneumoniae* are indistinguishable with our current approach. Therefore, our approach reported the most likely species based on how well the reads clustered with the type strains of given *Streptococcus* species. Further sequence and biochemical functional characterization will be needed to confirm the identity of Streptococcus species. Three species of *RothiaR. dentocariosaR. mucilaginosa* and an untyped strain, were identified in most OS and AS samples but only in 2 RC samples. Three species of *Capnocytophaga* were identified, *C. gingivalisC. sputigena*, and an untyped strain. Several *Leptotrichia* species were found in OS and AS samples. The most commonly found *Leptotrichia* species in our samples was *L. wadei*. Others included *L. buccalisL. goodfellowiiL. hofstadiiL. shahii*, and *L. trevisanii*. It appears that for individuals harboring *Leptotrichia*, several species were present at once. There is only one species of *Abiotrophia *(*A. defective*) known to be associated with oral samples and BLAST search of our sequences against the CORE Oral Database confirmed that all of them matched *A. defectiva*. In addition to the differentially distributed genera, for genera that have more than 1000 combined reads from all samples, we listed the species breakdown in Additional file 5.

### OTU analyses revealed different levels of species diversity in the 3 sites

RDP Classifier results provided us with an overview of the taxa that were present in our samples at the genus level. This approach, however, has a few limitations. First, approximately 4% of the sequences could not be assigned to a genus so they were not included in the previous analysis. Second, the granularity of a named taxon is arbitrary and often based on gross morphologies, chemical tests, and arbitrary sequence similarity cutoffs so some taxa contain much more divergent organisms than others. Third, the sequencing coverage is different for each sample so it is necessary to normalize the datasets before one can compare across samples. We, therefore, complimented the RDP Classifier taxonomic results with operational taxonomic unit (OTU) analysis using Mothur software package [28]. OTUs were constructed based on sequence similarity of the 16S rRNA genes and the algorithm we used established an upper bound for the distances among all sequences within an OTU.

**Figure 3:**
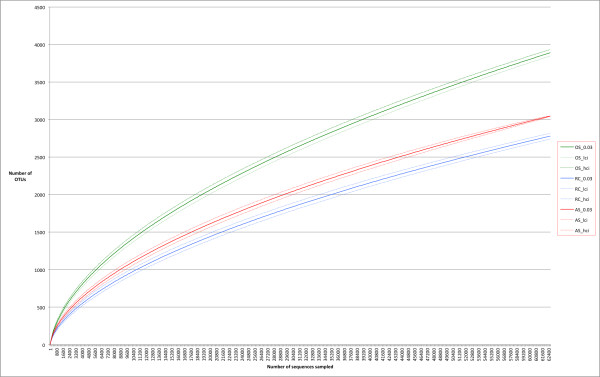
**Rarefaction curves by site (3% identity cutoff) – the dotted lines show the 95% confidence interval of each curve.** Note that there are no overlapping curves suggesting that the levels of diversity are significantly different among the sampling sites. (green = OS, blue = RC, and red = AS).

**Figure 4:**
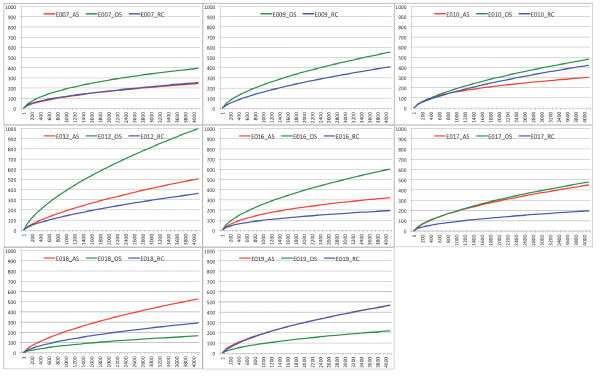
Rarefaction curves showing the “species” (OTUs at 3% cutoff) diversity of each sampling sites for each subject capped at the lowest sequencing coverage (4,200 reads).

We started with a total of 231,519 sequences binned by samples as in the RDP analyses. After applying quality filters as specified in the methods section, 227,892 (98.4%) sequences passed the initial filters. We identified 55,641 unique sequences from the dataset. After a round of pre-clustering to remove rare sequences that were likely to have arisen from sequencing errors, 34,723 sequence clusters were generated [29]. These unique sequences were further clustered at various distance cutoffs roughly corresponding to the different taxonomic ranks to produce OTUs. The total numbers of OTUs at various cutoffs were as follow: 17,287 OTUs at 99% sequence identity, 8,196 OTUs at 97% identity, 5,417 OTUs at 95% identity and 2,550 OTUs at 90% identity. Rarefaction curve, which provides a standardized measure of intra-sample (α) diversity, allows one to compare the organismal diversity across samples. Figures 3 shows the rarefaction curve of each sampling site. The results showed that the microbiota diversity was the highest in OS samples followed by the AS samples then by the RC samples, confirming the results obtained above based on known taxa. This result fits well with the hypothesis that root canal system and periapical abscess are more restricted environments than the healthy oral cavity so fewer species of bacteria are found there. However, we could not exclude the possibility that the larger sampling area in our oral sample contributed to the observed higher diversity. We sampled more widely in the oral cavity in order to demonstrate that oral cavity is the likely source of bacteria for root canal infections. Based on our analysis, even at the high sampling coverage that we have achieved, we still have not reached sampling saturation (flattened rarefaction curves) with OTU clustering cutoff of 3%. The richness (number of species) in our samples was on par with previous 454 pyrosequencing based surveys and was significantly higher than results reported from other low resolution or low throughput molecular techniques (hybridization, restriction-digestion or denaturing gradient gel electrophoresis, Sanger sequencing, and microarray). The simultaneous sampling of three sites in one individual undergoing root canal procedures allows direct comparison of the microbiota diversity of the normal vs. diseased oral sites. At a uniform sampling depth of 4200 reads (lowest number of reads obtained from one sample), we detected, on average, 486 OTUs in one individual’s OS samples and 325 OTUs and 403 OTUs for RC and AS samples, respectively. Figures 4 shows the rarefaction curves of “species” diversity (3% cutoff) for each individual. While there are some discrepancies for which the reasons are not known, the majority of the individuals also show the same species diversity pattern for the 3 sampling sites.

### Abundant OTUs were often found in all 3 sampling sites

**Figure 5:**
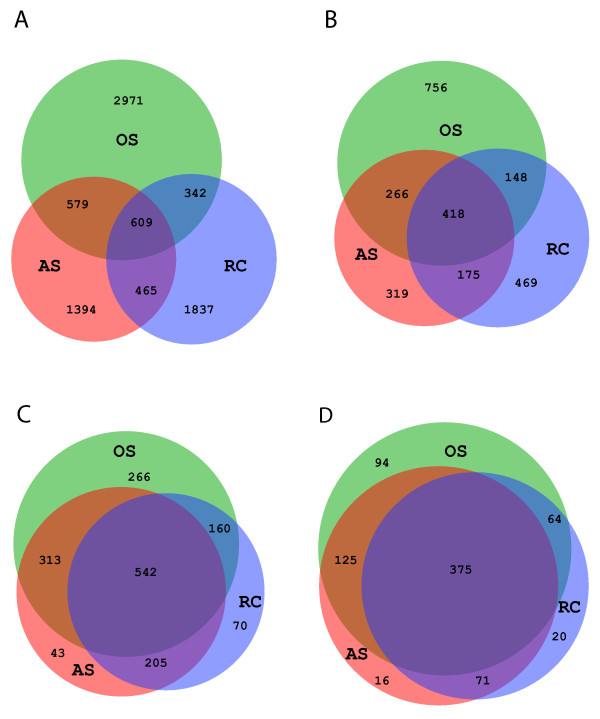
**(A-D) Venn diagram of shared OTUs among the 3 sampling sites (green = OS, blue = RC, and red = AS)**. Panels: **A**) all OTUs at 3% and **B**)10% cutoffs, **C**) abundant OTUs at 3% and **D**) 10% cutoffs. Overlapping regions were drawn to scale and the number of shared and unique OTUs listed.

To find out if different sampling sites share the same OTUs, we examined the number of shared OTUs between OS, RC, and AS samples. Figures 5 panels A and B show the number of shared OTUs at 3% and 10% cutoffs (97% and 90% sequence identities, respectively). The proportions of OTUs shared appear to be low at both cut-offs. However, upon closer examination, the shared OTUs were mostly the more abundant OTUs while the site-specific OTUs were mostly the low abundance OTUs. 78.6% of the sequences (178,880/227,526) belonged to OTUs (97% identity cutoff) that were detected in all 3 sampling sites. When OTUs with fewer than 5 sequences (i.e. rare OTUs) were excluded from the analysis, it became clear that most of the abundant OTUs were shared between two or among all three sampling sites (Figures 5 panels C and D). We therefore can conclude that site-specific OTUs were mostly low abundant species. It is also interesting to note that the OS samples had the most site-specific OTUs. This result suggests that there is a continuous microbial transformation in the transition betweenthe healthy oral cavity and the diseased root canal and periapical abscess sites. The relative abundances of these shared species, however, shift from one site to another as shown in the previous section and below. Taken together, these results suggest that the key species associated with diseased sites may be more rare species. Alternatively, changes in relative abundance and the presence of opportunistic pathogens in restricted oral sites may contribute to the diseases.

Microbiota community structures suggest that root canal and abscess samples were more similar to each other than to oral samples

**Figure 6:**
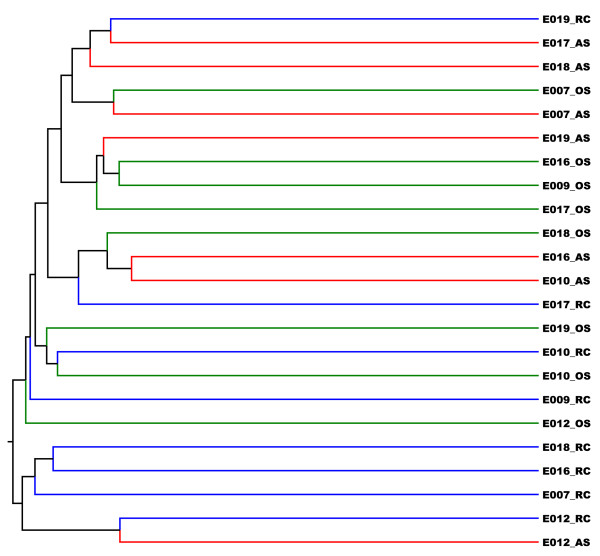
**JClass sample tree generated using Mothur.** (green = OS; blue = RC; red = AS).

**Figure 7:**
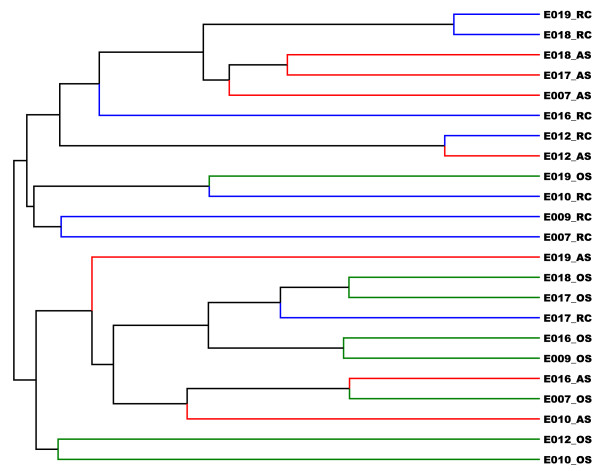
**Theta-YC sample tree generated using Mothur.** The top branch consists of mostly root canal samples (RC) while the bottom branch consists of mostly oral samples (OS). (green = OS; blue = RC; red = AS).

To further compare the microbiota profiles of the different individuals and different sites, we performed unsupervised clustering of the 23 samples using Jaccard dissimilarity index and Yue and Clayton (Y&C) dissimilarity index. The Jaccard index derives the dissimilarity measures based on the presence and absence of OTUs so it is a measure of shared community membership. The Y&C index takes relative abundances of shared OTUs into account so it is a measure of community structure. At the 0.03 cut-off, we did not see obvious clustering by sites or by individuals using Jaccard index. When we increase the cutoff to 0.05 and higher, we observed that some RC samples clustered together while OS and AS samples were intermixed (Figures 6). This result suggested that RC samples have more distinct taxa compared to OS or AS samples. This is in agreement with the observation that there are overall more shared OTUs between AS and OS samples (Figures 5) than between RC and OS samples. Using Y&C index, we observed two major branches in the dendrogram (Figures 7). One branch contains mostly AS and RC samples while the other contains mostly OS and some AS samples. This branching pattern was consistent regardless of the cutoff used (from 0.03 to 0.15). Using parsimony (P) test, we also confirmed that the clustering pattern within the dendrogram is statistically significant (p<0.02) [30]. To verify that AS and RC microbiota profiles were more similar to each other than to the microbiota profile of OS samples, we pooled the reads from same sampling sites and carried out unsupervised clustering with Jaccard and Y&C indices. The AS and RC microbiotas cluster together and were more similar to each other than to the OS samples. These results support the hypothesis that oral cavities provide the microbial sources that feed into the root canal infection sites.

### Certain bacterial species were associated with diseased sites

We reported the over and under-represented genera in the different sample sites above. Since a genus can contain both pathogenic and non-pathogenic species, we re-examined our data at a finer resolution of OTUs at 1% cutoff (i.e. each OTU contains sequences that were 99% identical to all other sequences in the same OTU). To examine which OTUs were over or under-represented in one sampling site compared to another, we used ANOVA tests on normalized and arcsine transformed OTU count data as we did with the RDP Classifier results. Of the 17287 OTUs, only 24 of them were significantly different in one site compared to another at p-value < 0.01. These 24 differential OTUs correspond to the genera *Bacteroides, Granulicatella, Collinsella, Atopobium, Dialister, Diaphorobacter, Lachnospiraceae incertae sedis, Moryella, Prevotella, Streptococcus, Veillonella,* unclassified Bacteroidales and unclassified Clostridiales based on Mothur assignments. The OTUs assigned to Lachnospiraceae were labeled as incertae sedis (uncertain placement) since the membership to this taxon is still in flux. Using BLAST search against NCBI 16S Microbial database, we found that this OTU matched several different species in the Lachnospiraceae family equally well but at relatively low sequence identity (88-89%). More than one OTU were assigned to *Prevotella*, *Veillonella*, *Streptococcus*, and *Bacteroides* suggesting that these species may be preferentially associated with certain sites. With the large number of OTUs and small sample size, even with a p-value of <0.01, the differential OTUs can still occur due to chance. We therefore also looked at OTUs at 3% cutoff to see which of the genera observed above also show differential distributions at the more relaxed cutoff. Besides *Dialister* and *Collinsella*, all the other genera were also found in differential OTUs at 3% cutoff. At 3% cutoff, an additional OTU belonging to the genus *Fusobacterium* also showed higher relative abundance in diseased samples than in oral samples (p = 0.002). Several *Fusobacterium* OTUs at 1% cutoff also showed higher relative abundances in diseases samples albeit with higher p-values (>0.01 and < 0.05). Many of the differential OTUs at 3% cutoff represented a superset of the OTUs at 1% cutoff so the datasets were not independent. However, some differential OTUs in the 1% cutoff dataset were not represented in the 3% cutoff set and vice versa. Yet, they corresponded to the same genera or species. Additional file 6 summarizes the results from the ANOVA tests.

We searched the representative sequences of these differential OTUs (at 1% cutoff) against the HOMD and NCBI 16S databases using NCBI-BLAST to identify the likely species designations. Eight of the 24 OTUs matched known oral species with greater than 99% identity and can be reasonably assigned to these species. In these cases, both HOMD and NCBI BLAST searches returned the same species as the top hits with the second hits at significantly lower sequence identities. Others (16 out of 24), however, appear to be novel species or more divergent isolates with lower sequence similarity to known species in both NCBI and HOMD databases (see Additional file 6 for BLAST results). We used the high stringency cutoffs for both OTU clustering (>99% identity) and database matches (>99% identity over the length of the alignment) to achieve an overall sequence identity of 98% (1% potential mismatches from each step) in order to be reasonably certain about the species assignment. We also manually examined the BLAST results to ensure that there were no hits to other species at comparable similarities. The OTUs with positive assignments belonged to *Granulicatella adiacens, Eubacterium yurii, Prevotella melaninogenica* (3 different OTUs belong to this species), *Prevotella salivae, Streptococcus mitis* and *Atopobium rimae*(Additional file 6). Other OTUs with lower similarity matches to the databases are also listed in Additional file 6 for reference. While the ultimate identity of these OTUs need additional molecular and/or cultural isolations to verify, we report them here to facilitate future studies.

We were particularly interested in OTUs that were over-represented in infected root canals or periapical abscess compared to oral cavity since these may represent organisms that were associated with the infections. These include *Granulicatella adiacens (*related to *Streptococcus* genus*)Prevotella melaninogenicaPrevotella salivaeStreptococcus mitisVeillonella parvula* related OTUs, a *Streptococcus sanguinis* related OTU and *Lachnospiraceae* related OTUs. It’s worth noting that in all these cases, multiple OTUs in the same species showed the same directions of change. For example, all *Prevotella melaninogenica* OTUs that were differentially distributed showed higher relative abundance in root canal and abscess samples. Also, many of these organisms (e.g. *G. adiacensS. mitisP. melaninogenica*, and *V. parvula*) have been reported as opportunistic pathogens in diseases or were associated with diseases [31-34]. The literature thus supports our ability to identify known pathogens using a 16S rRNA amplicon sequencing approach, which look at the entire microbiota of each site with much higher depth coverage and much lower selection biases than previous studies. It is possible that only sub populations of these reported species are associated with diseases. Therefore, it is important to follow-up the association study with more detailed genomic or pan-genomic studies to gain better understanding of these opportunistic pathogens [35]. Our results present the necessary species identification to pursue these experiments.

### Certain bacterial OTUs were only found in systemic infection

Root canal infections can lead to spreading systemic infections characterized by fever, lymphadenopathy, and /or fascial space involvement and accompanied by elevated serum cytokines and acute phase proteins. We hypothesized that certain bacteria were associated with systemic infection andthat shifts in microbiota not only reflects the microbial adaptations to different environmental niches (especially from a more oxygenated oral cavity to a more anaerobic environment (root canal and abscess) but the shifts may also reflect the disease progression. Comparing systemic-infection samples and localized-infection samples for each sampling site using paired t-tests, we identified some OTUs in each site that were differentially distributed in systemic infections vs. localized infections (with p-values < 0.05) (Additional file 7). Because of the small sample sizes, we reported differentially distributed OTUs that were only found in systemic infections and were not found in localized infection samples. These typically have very low p-values from paired t-tests as expected. Notably, many of these OTUs belong to the genera, *Prevotella, Fusobacterium, Actinomyces, Veillonella,* and *Streptococcus.* Further isolation and identification of these OTUs will be necessary to study their roles in spreading systemic infections. Larger sample size will also enable us to confirm that these OTUs are indeed specific to systemic infections.

## Conclusions

In this study, we obtained microbiota samples from normal and diseased oral sites from the same individuals and sequenced the 16S rRNA gene fragments using a next generation high-throughput sequencing platform to obtain on average 10,000 sequences per sample. Using a variety of bioinformatics and statistical approaches, we demonstrated that the microbiotas from normal and diseased oral sites are distinct. First, the overall oral cavity microbiota was more diverse than the microbiota from infected root canals and abscesses. This is likely to be a function of the wider array of oral cavity microenvironments we sampled. Second, the relative abundance of different taxa shifted from one site to another. Root canal and abscess samples appeared to be dominated by anaerobic organisms as expected while oral microbiota had a mix of aerobic, facultative and anaerobic organisms. The microbiota of the root canal system and that of periapical abscess were found to be more similar to each other than to oral samples based on microbiota community structure but the observation is less clear based on community membership. However, the abundant taxa often were found across multiple sites suggesting that while micro-environments and different microbial communities exist in the mouth, bacteria can disseminate from the oral cavity, into the root canal system and the periapical abscess. We applied normalization and arcsine transformation to our taxonomic count data so standardized statistical tests could be applied to the samples. Using paired t-tests, we identified known taxa and novel OTUs that were differentially distributed in the different sample sites.

This is the first study that we are aware of which used high-throughput next generation sequencing to compare samples from normal and diseased oral tissues from the same individuals. Using novel algorithm, SpeciateIT, and with careful manual inspection of the BLAST alignments and results, we were able to confidently assign some of the OTUs down to the specieslevel. We reported some of the known opportunistic pathogens such as *G. adiacens* to be associated with diseased oral sites. We also reported several novel OTUs, which will require further characterization, to be associated with diseased sites. While we acknowledge the limitation of using only fragments of 16S rRNA gene for species assignment, we believe we have demonstrated due diligence in setting very strict criteria and by manually inspecting the BLAST search results from multiple databases. We found that the BLAST search results from different databases to be consistent as long as the query and the database hits were > 99% identical. Moreover, we demonstrated that below this cutoff, search results can differ due to coverage biases of the different databases. Most of the similar studies to date stopped at the genus level which is insufficient to provide the necessary information to plan the next experiments since both pathogenic and nonpathogenic species can fall within the same genus. However, we still need to caution the readers that the assignments are based on fragments of 16S genes and that pyrosequencing has higher error rates than traditional Sanger sequencing. Despite our effort to reduce errors by using several filters, there are most likely undetected sequencing errors in our reads. Therefore, for rare species and species such as *Streptococcus* where identification based on 16S gene is challenging, functional characterization should be performed in follow-up studies. This study, like other microbiome studies, can certainly benefit from larger sample size and time-series study design. However, the difficulty in identifying healthy patients with healthy oral cavity except for an endodontic acute infection, and for these patients not to be already on antibiotics needs to be recognized. Time-series studies will provide more information on the microbiota dynamics. It is possible that shift in microbiota can occur in infected sites as the disease progresses and such shift can be very informative for prognosis and for design of treatment regiments. However, temporal study designs may place undue burden on the patients so careful design will be needed. In summary, this study provided an in-depth characterization of the microbiota of the normal oral tissues and diseased root canal sites using one individual as his or her own baseline control. Functional metagenomic studies and detailed molecular analyses of some of the opportunistic pathogens identified in this study are being planned for the future.

## Methods

### Patient and samples

All patient-related procedures used in this study conformed to protocols approved by the Institutional Review Board of the University of Maryland School of Medicine. Eight patients (5 with systemic infections and 3 with localized infections) were included according to the following criteria: male or female patients referred for endodontic treatment (n = 6) or extraction (n = 2) for pulp necrosis and acute periapical abscess, and willing to have a 10ml tube vial of blood drawn, ages of 12 and older, availability of panoramic radiographs and information to determine the presence of other endodontic infections or periodontal disease. Systemic infections were cases in which the patient had fever, lymphadenopathy and/or fascial space infection. Localized infections had a localized swelling, but none of these other signs. Four of the 8 teeth included were molars. Exclusion criteria were: smokers, women of child-bearing age who indicated that they were pregnant, patients taking antibiotics, patients with systemic diseases such as diabetes mellitus, cardiovascular disease, atherosclerosis, and history of stroke, HIV/AIDS, rheumatoid arthritis or any other systemic disease that compromises the immune system, chronic periodontitis defined as clinical attachment loss ≥ 6 mm or probing depth ≥ 5 mm in two sites in the mouth, frank caries with cavitation, oral candidiasis, ulceration or other infections or neoplasia, teeth with open apex, teeth that fractures during extraction, or teeth with previous endodontic treatment. Most of the teeth included were intact but had been devitalized by traumatic injuries or had previous adequate restorations underneath which the pulp became necrotic.

Patients were provided routine emergency treatment for the tooth involved, which included either extraction of the tooth or root canal debridement, together with systemic antibiotics in the systemic infection group. Following satisfactory local anesthesia, recommended treatment: either an extraction or root canal therapy was performed according to the plan for emergency management.

Three specimens were obtained from each patient at the same time. The first specimen (OS) was a Total Oral Sample. It was collected from the subjects by rubbing 3 fine paper points against patient’s cheek mucosa, lateral boarder of the tongue and supragingival plaque at the contra lateral side away from the affected tooth. The second specimen (AS) was an Abscess Aspiration Sample. The area of the swelling was aspirated with a syringe that had a 16 gauge needle, and expressed into a sterile vial. The third specimen (RC) was a Root Canal Sample collected from the root canal space of the diseased tooth. For teeth treated endodontically, bacterial samples were collected according to a protocol previously used by our group [15,36]. After rubber dam isolation, the tooth surface was disinfected with 30% hydrogen peroxide, followed by 5% tincture of iodine, 5.25% sodium hypochlorite and finally 5% sodium thiosulfate to inactivate the halides. The pulp chamber was accessed with #4 round carbide bur followed by an endo Z bur. Canals were identified under a surgical operating microscope. Three Fine paper points were placed in each canal to collect fluids from the canal space. If purulence or serous fluid was present in the canal, this was directly sampled with three fine size paper points followed by a sterile file to the working length, as indicated by an apex locator and then aseptically separated into the sampling vial. In multicanaled teeth, one paper point sample was obtained from each canal unless the canals were very calcified, in which case sampling of the canal root with the largest periapical lesion and the largest canal was sampled. If the canal was dry, sterile saline was added, the biofilm disrupted with a file, and the file together with three paper points were similarly sampled. The file and paper points were placed insterile, DNA- and RNA-free vials containing 1.5mL of filter sterilized 10mM Tris-HCl, (pH 8.0). Then root canal treatment was completed for these patients. For patients needing extraction, after extraction, the retrieved tooth was disinfected completely with 30% hydrogen peroxide, 5% tincture of iodine, 5.25% sodium hypochlorite and 5% sodium thiosulfate. Tooth then was sectioned at the cemento-enamel junction (CEJ) with a taper fissure bur, leaving the root intact. Then the same protocol of canal sampling that was used for endodontic treated patients was followed for the sectioned root fragments.

### DNA extraction

For DNA extraction, the paper filter used to collect the specimen was transferred to a DNA/RNA-free sterile tube, and 1ml of phosphate-buffered saline was added to the sample. Cell lysis was initiated by adding 10 μl Proteinase K (at 20mg/ml) and 50 μl 10% SDS, followed by incubation at 60°C for 30 minutes. After 30 minute incubation at 60°C, each sample was further lysed by addition of 200 ul of cell lysis buffer (100mM Tris-HCl pH 7.4, 20 mM EDTA, 5M guanindine isothiocyanate). The samples were then disrupted by bead beating, which was performed in a FastPrep instrument FP120 at 6.0 m/s for 40 sec using 0.1 mm silica spheres (QBiogen Lysis Matrix B). The resulting lysate was transferred to a clean tube and precipitated with a 1/10 volume of 3M NaOAc, 3 volumes of cold ethanol (100% ethanolkept at -20°C) and 2ul of pellet paint, left overnight at -20°C, then spun for 30 minutes, followed by a 70% ethanol wash twice and re-suspended in Tris EDTA. Negative extraction controls were performed to ensure the samples were not contaminated by exogenous bacterial DNA during the extraction process. The DNA concentrations in the samples were measured using the Quant-iT PicoGreen dsDNA assay kit from Molecular Probes (Invitrogen).

### Pyrosequencing of barcoded 16S rRNA gene amplicons

Universal primers 27F and 338R were used for PCR amplification of the V1–V2 hypervariable regions of 16S rRNA genes. The 338R primer included a unique sequence tag to barcode each sample. The primers were as follows: 27F-5′-GCCTTGCCAGCCCGCTCAGTC**AGAGTTTGATCCTGGCTCAG**-3′ and 338R-5′-GCCTCCCTCGCGCCATCAGNNNNNNNNCAT**GCTGCCTCCCGTAGGAGT**-3′, where the underlined sequences are the 454 Life Sciences FLX sequencing primers B and A in 27F and 338R, respectively, and the bold letters denote the universal 16S rRNA primers 27F and 338R. The 8-bp barcode within primer 338R was denoted by 8 Ns. Using 96 barcoded 338R primers [listed in Additional file 8; originally published in [27]] the V1–V2 regions of 16S rRNA genes were amplified in 96-well microtiter plates using AmpliTaq Gold DNA polymerase (Applied Biosystems, Carlsbad, CA) and 50 ng of template DNA in a total reaction volume of 50 μL. Reactions were run in a PTC-100 thermal controller (MJ Research, St. Bruno, QC) using the following cycling parameters: 5 min of denaturation at 95 °C, followed by 20 cycles of 30 s at 95 °C (denaturing), 30 s at 56 °C (annealing), and 90 s at 72 °C (elongation), with a final extension at 72 °C for 7 min. Negative controls without a template were included for each barcoded primer pair. The presence of amplicons was confirmed by gel electrophoresis on a 2% agarose gel and staining with SYBRGreen. PCR products were quantified using a GelDoc quantification system (BioRad, Hercules, CA) and the Quant-iT PicoGreen dsDNA assay. When possible, equimolar amounts (100 ng) of the barcoded PCR amplicons were mixed in a single tube. Amplification primers and reaction buffer were removed from each sample using the AMPure Kit (Agencourt, Danvers, MA). The purified amplicon mixtures were sequenced by 454 FLX Titanium pyrosequencing using 454 Life Sciences primer A by the Genomics Resource Center at the Institute for Genome Sciences, University of Maryland School of Medicine, using protocols recommended by the manufacturer as amended by the Center.

### Sequence processing and analysis

Sequences were binned and trimmed based on the sample-specific barcodes using a custom perl script and Mothur with the following criteria: (i) sequence length > 199nt, (ii) sequence length <450nt, (iii) no ambiguous base in the sequences, and (iv) a maximum of 8 homopolymers. Moreover, sequences that failed to align within positions 1044 and 6333 of the Silva reference alignment provided by Mothur website were also discarded. Initial taxonomicclassificationswere done using RDP Classifier ver. 2.2 [22] at confidence threshold of 0.5 against the RDP databasesupplied with the program. Additional taxonomic assignments were done using SpeciateIT program developed at the Institute for Genome Sciences [27]. Operational taxonomic units (OTUs) were determined using Mothur ver. 1.17 by (i) alignment to the SILVA 16S rRNA database [37], (ii) pre-clustering to reduce pyrosequencing errors [29], (iii) calculating the pair-wise distances of the sequences using Mothur’s default settings, and (iv) hierarchical clustering with default settings and extracting clusters (i.e. OTUs) at various cut-offs roughly corresponding to conventional taxonomic ranks [28].To determine how similar the samples from different individuals and different oral sites were to each other, the presence and absence of OTUs in each of the samples and the relative abundances of OTUs in each of the samples were used to cluster the samples using Jaccard index and Theta-YC dissimilarity index, respectively. Rarefaction curves were plotted for each sample and for each oral sites using Mothur. Shared OTUs among the three oral sites were plotted as Venn diagrams using all OTUs and abundant OTUs only. BioVenn web application is used to plot the Venn diagrams with some post-editing to improve legibility [38]. Abundant OTUs were defined as OTUs with at least 5 sequences across all samples. Each OTU was assigned a taxonomic label down to the genus level using Mothur’s classification algorithm against RDP database as reference. Representative sequences of OTUs that were significantly differentially distributed among the different sampling sites were further searched against HOMD (v. 10.1) and NCBI 16S databases (Dec 2011) using default parameters for possible species identification. All the search results were manually inspected and database matches that exceed 99% identity over the length of the aligned region were reported as possible species matches.

Differentially distributed taxa (RDP Classifier results) and OTUs (Mothur results) were identified as following based on a method we developed and published [26]. Briefly, to correct the sampling depth variations across samples and the different variances of the abundance species compared to those of the rare species, we calculated the relative abundance of each taxon/OTU within each sample and applied arcsine transformation to the proportions [26]. The transformation helps to stabilize variances in abundant vs. rare taxa. Then, using ANOVA and paired t-tests, we compared taxa/OTUs from one site to another to identify taxa/OTUs that were differentially distributed in one site with respect to another. By using paired t-tests and sampling multiple sites within the same individuals, we can establish the baseline normal microbiota for each individual and use that as a reference to analyze the microbiota of diseased sites. This approach allowed better comparisons of the oral microbiota from normal and diseased samples and better detection of consistent trends since each individual harbors a variety of oral microbiota and was his or her best control.

## Abbreviations

OTU, Operational taxonomic unit; OS, Oral cavity swab samples; RC, Root canal samples; AS, Periapical abscess samples.

## Competing interests

The authors declare no competing interests.

## Authors’ contributions

WWLH, LL, CMFL, and AF conceptualized and designed the study. LL and AF collected the patient samples. CJ processed the samples. WWLH performed the sequence and data analyses. WWLH, ZL and AF analyzed the results. WWLH and AF wrote the manuscript and all authors reviewed and edited the manuscript. All authors read and approved the final manuscript.

## Supplementary Material

Additional file 1 **Bar graph showing the number of reads from each sample.** (DOCX 337 kb) Click here for file

Additional file 2 **Rarefaction curves by sample sites for each subject.** (XLSX 274 kb) Click here for file

Additional file 3 **Relative abundance (before_arcsine) and transformed abundance (after_arcsine) of all genera found in each of the samples.** (XLSX 64 kb) Click here for file

Additional file 4 **Differentially distributed genera (p<0.05 are listed, p<0.01 are highlighted) detected using ANOVA and paired-t tests of the transformed abundance data.** The original counts for each genus are listed to show that most of these organisms are not dominant organisms in the samples. (XLSX 56 kb) Click here for file

Additional file 5 **SpeciateIT results.** (XLSX 2243 kb) Click here for file

Additional file 6: **Differentially distributed OTUs (1% cutoff) annotated with Mothur k-mer Classifier and BLAST searches against HOMD and NCBI databases.** (XLSX 414 kb) Click here for file

Additional file 7 **Paired-t tests and ANOVA tests of systemic infection samples vs. localized infection samples.** (XLSX 16 kb) Click here for file

Additional file 8 **Table S2.** Barcoded PCR primers used for the amplification of 16S rRNA genes. (XLSX 42 kb) Click here for file
