# Supplementary figures and images for: Microbial transformation from normal oral microbiota to acute endodontic infections
Source: BMC Genomics. 2012 Jul 28;13:345. doi: 10.1186/1471-2164-13-345 (PMC3431219; doi:10.1186/1471-2164-13-345)

Additional File 1: Number of reads from each sample.


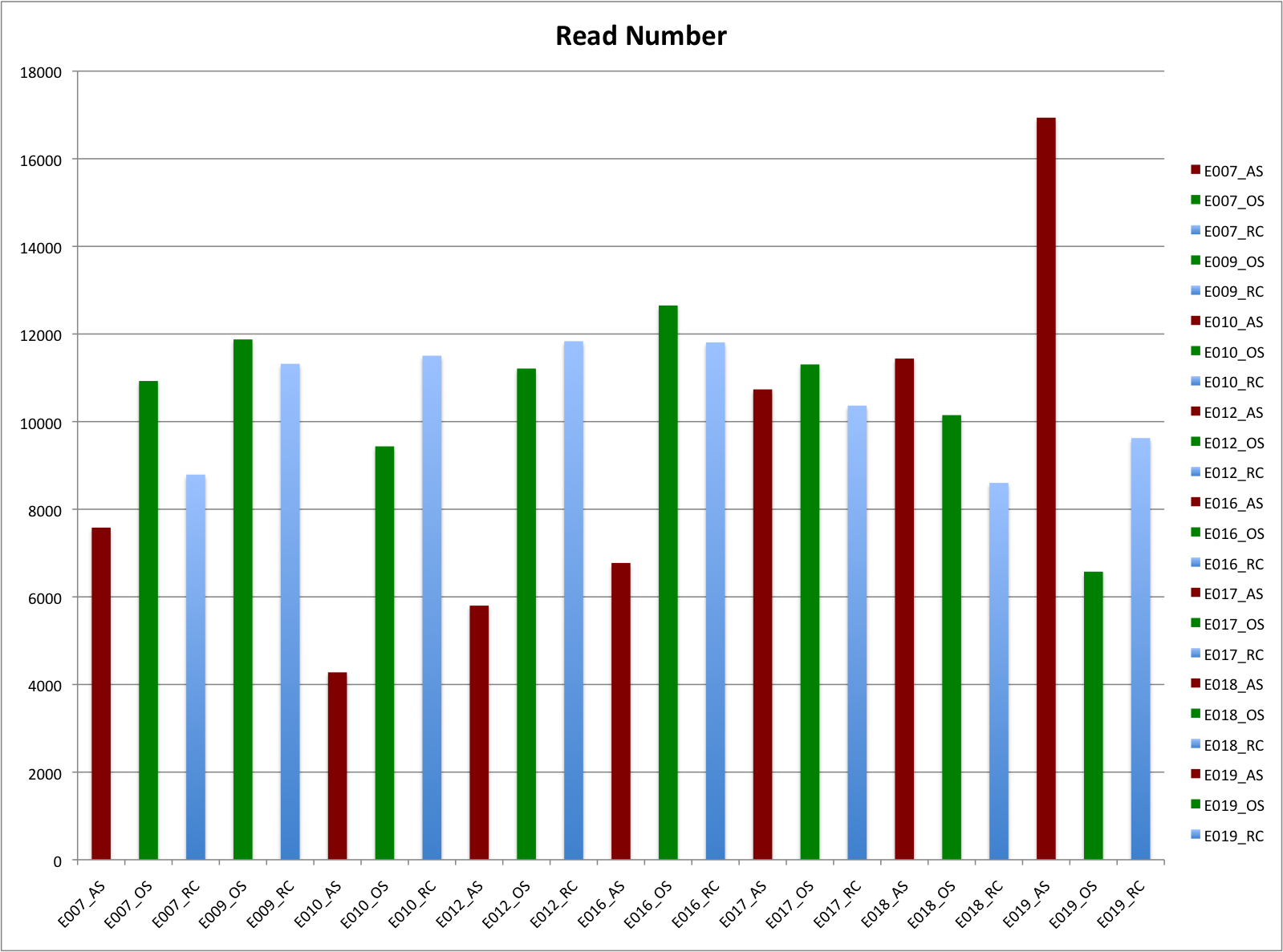

Supplement: Additional file 1 — Bar graph showing the number of reads from each sample. (DOCX 337 kb) [file 1471-2164-13-345-S1.docx]
